# Impact of Hyperbaric Oxygen Therapy on Cognitive Functions: a Systematic Review

**DOI:** 10.1007/s11065-021-09500-9

**Published:** 2021-04-13

**Authors:** Anna B. Marcinkowska, Natalia D. Mankowska, Jacek Kot, Pawel J. Winklewski

**Affiliations:** 1 Applied Cognitive Neuroscience Lab, Department of Human Physiology, Medical University of Gdańsk, Tuwima Str. 15 80-210, Gdańsk, Poland; 2 2nd Department of Radiology, Medical University of Gdańsk, Gdańsk, Poland; 3 National Centre for Hyperbaric Medicine, Institute of Maritime and Tropical Medicine in Gdynia, Medical University of Gdansk, Gdańsk, Poland

**Keywords:** Neuropsychology, Cognition, Hyperbaric oxygen therapy

## Abstract

Hyperbaric oxygen therapy (HBOT) is a modality of treatment in which patients inhale 100% oxygen inside a hyperbaric chamber pressurised to greater than 1 atmosphere. The aim of this review is to discuss neuropsychological findings in various neurological disorders treated with HBOT and to open new perspectives for therapeutic improvement. A literature search was conducted in the MEDLINE (via PubMed) database from the inception up 10 May 2020. Eligibility criteria included original articles published in English. Case studies were excluded. Full-text articles were obtained from the selected studies and were reviewed on the following inclusion criteria (1) performed cognitive processes assessment (2) performed HBOT with described protocol. Two neuropsychologists independently reviewed titles, abstracts, full texts and extracted data. The initial search retrieved 1024 articles, and a total of 42 studies were finally included after applying inclusion and exclusion criteria. The search yielded controversial results with regard to the efficiency of HBOT in various neurological conditions with cognitive disturbance outcome. To the best of our knowledge this is the first state-of-the art, systematic review in the field. More objective and precise neuropsychological assessment methods are needed to exact evaluation of the efficacy of HBOT for neuropsychological deficits. Future studies should widen the assessment of HBOT effects on different cognitive domains because most of the existing studies have focussed on a single process. Finally, there is a need for further longitudinal studies.

## Introduction

Hyperbaric oxygen therapy (HBOT) is a modality of treatment in which patients inhale 100% oxygen through a head tent, mask or endotracheal tube inside a hyperbaric chamber that has been pressurised to greater than 1 absolute atmosphere (ATA). HBOT is typically administered at more than one and less than three ATA and induces a state of increased pressure and hyperoxia that cause mechanical and physiologic effects.

HBOT has been recommended for various conditions for more than 40 years (Grim, 1990; Leach et al., 1998). Initially, it was used to treat decompression sickness in divers. However, over the years its far-reaching potential has been recognised, and it has been approved for a variety of purposes, including carbon monoxide (CO) poisoning, decompression sickness and gas embolism, problematic wound healing, delayed radiation injuries, sudden deafness and other conditions as indicated by evidence-based medicine (Mathieu et al., 2017; Moon, 2019). Although controlled clinical trials are limited, the rational basis of HBOT as well as good clinical results have gradually increased the use of HBOT for neurological disorders linked with cognitive disturbances. Neurological disorders as well as conditions related to central nervous system (CNS) damage may present with a variety of neuropsychological symptoms, such as impairment of memory and learning processes, attention and visuo-spatial functions, language processes and executive dysfunctions.

The neuroprotective and therapeutic effects of neuropsychological deficits provided by HBOT have been established in experimental animal and human models, although they remain controversial. In this review, we will summarise the existing results of HBOT usage in several neurological states, its current understanding and opinions for future studies. To the best of our knowledge this is the first state of the art, systematic review in this topic.

To understand the role of HBOT in neurological disorders, a basic knowledge of cerebral metabolism, cerebral blood flow and the neurophysiology of the brain is essential. The physiological basis of HBOT is the gas laws. While breathing air at atmospheric pressure, most of the oxygen is bound to haemoglobin. In this situation, blood haemoglobin is saturated with oxygen (at approximately 97%) and working at near maximum capacity with a small amount of oxygen dissolved in the blood plasma compartment. If the percent of inhaled oxygen – or the pressure at which oxygen is breathed – is increased, more oxygen will dissolve into the blood plasma. In HBOT conditions, the fraction of inspired oxygen and partial pressure of oxygen increases, supersaturating the blood with oxygen. Inhaling 100% oxygen at 3 ATA increases mean arterial oxygen tension from approximately 100 mmHg in normobaric conditions to 2000 mmHg and the amount of oxygen delivery to the tissues from 3 to 60 mL of oxygen per litre of blood (Jain, 2017). Supersaturation of the blood to this degree supports resting tissue oxygen requirements without a necessary contribution from haemoglobin carriage. Hence, HBOT is useful in diseases in which haemoglobin function is limited, such as CO poisoning or ischaemia (Mathieu, 2006a). The excess oxygen is carried in solution and it can diffuse to areas where red blood cells cannot reach. This elevation in the partial pressure of oxygen in tissue mediates the therapeutic benefits of HBOT.

Enhanced oxygen supply and increased pressure result in a wide variety of pathophysiological mechanisms. Thus, HBOT is believed to diminish neuroinflammatory responses, blood–brain barrier permeability and apoptosis while positively impacting neurogenesis, neuronal and axonal integrity and synaptogenesis (reviewed by Daly et al., 2018; Fischer et al., 2010). All of these effects may potentially influence patients’ cognitive functioning. Nevertheless, direct links are very difficult to establish. Recent advances in radiology and medical imaging, in particular diffusion tensor imaging (DTI) and advanced perfusion models, could potentially fill the gap and provide better understanding of the interdependence among white matter structure, cerebral blood flow and cognition (Chen et al., 2010; Tal et al., 2017).

Brain metabolism generates large amounts of reactive forms of oxygen. There is an excessive literature demonstrating that augmented oxygen reactivity is associated with deteriorated performance in cognitive tasks (Bhatt et al., 2020; Kandlur et al., 2020). At the same time, however, reactive oxygen species may also play a role in functional and structural modifications indispensable for synaptic plasticity (Massaad & Klann, 2011). In particular reactive forms of oxygen seem to be involved in regulation of N-methyl-d-aspartate (NMDA) receptors (Betzen et al., 2009), calcium (Ca^2 +^) channels (Huddleston et al., 2008), potassium channels (Gong et al., 2000) and Ca^2 +^ /calmodulin kinase II (Shetty et al., 2008) functioning. Hydrogen peroxide promotes ryanodine receptor redox modifications in hippocampal neurons (Kemmerling et al., 2007). Consequently, although oxygen reactive forms seem to be necessary for long term potentiation (the cellular substrate for memory), they are also implicated with deficient long term potentiation during aging (Hu et al., 2007) and in mouse models of Alzheimer Disease (AD) (Cai et al., 2008). HBOT schemes differ from study to study what may result in various exposures to oxidative stress. Therefore, it is difficult to predict whether beneficial or detrimental effects of reactive oxygen forms on cognition in particular study prevails.

The diversity and strength of innate repair mechanisms activated by HBOT are associated both with the elevated level of dissolved oxygen and the elevated pressure (Efrati & Ben-Jacob, 2014b). However, it remains unclear how oxygen and pressure relate to each other. For instance, it has been proposed that HBOT may act as a transducer to improve oxidative metabolism while subsequent normobaric oxygen therapy (100% oxygen, 1 ATA) may further enhance this effect (Rockswold et al., 2010, 2013). Exposure of rat brain slices to high pressures (5.3 ATA) typically shows an augmentation in the synaptic NMDA receptors response, usually followed by post-synaptic excitability modifications (Bliznyuk et al., 2020; Mor & Grossman, 2010; Mor & Grossman, 2007). HBOT and normobaric reoxygenation augment excitability and activate oxygen-induced potentiation in CA1 hippocampal neurons (Garcia et al., 2010b). Moreover, oxygen induced neural plasticity does not need modifications in excitatory synaptic transmission (Garcia et al., 2010a). When referring to experiments performed with the use of brain slices it should be kept in mind that it remains unclear whether the reported hyperexcitability, and which forms of increased neuronal excitability are a part of the learning process or, actually represent cellular manifestation of neuronal oxygen poisoning. The only experiments (performed by our team) on humans exposed to 1.4 ATA and 2.8 ATA HBO may suggest that actually oxygen related mechanisms affecting cognition and poisoning might be interrelated (Kot et al., 2015). The study was, however, performed on young males from the elite military Special Forces unit and therefore might not be representative for wider population.

Traditional view is that hyperoxia (below 2.8 ATA) diminishes brain perfusion (Di Piero et al., 2002; Kety & Schmidt, 1948; Omae et al., 1998) due to cerebral vasoconstriction caused by an interruption in nitric oxide-mediated basal relaxation of cerebral vessels (Bitterman & Bitterman, 1998; Oury et al., 1992). The topic has been extensively reviewed by our team (Winklewski et al., 2013). The existing dogma has been challenged by Micarelli et al. (2013). Authors investigated 7 healthy volunteers during acute exposure to HBO at 2.5 ATA (thus mimicking a possible therapeutic condition). The authors reported augmented brain perfusion in the left hemisphere with a relative cerebral blood flow increase in the neural networks encompassing dorsal and ventral attention pathways. Nevertheless, the links between changes in brain perfusion and cognition are complex and not fully understood. Quite insightful are data coming from the investigations of the impact of physical training on perfusion and cognition. It is generally believed that active lifestyle slowdown cerebral prefusion and cognitive decline associated with age (Boraxbekk et al., 2016; Jackson et al., 2016; Szalewska et al., 2017). However, Guiney et al. (2019) demonstrated that improvements in higher-order cognitive functioning in healthy older adults associated with habitual physical activity were not related to improved cerebrovascular functioning.

Hyperoxia and in particular HBOT exposure results in increased parasympathetic nervous system activity, bradycardia and dissociation between heart rate and broader cardiovascular system responses (Lodato & Jubran, 1993; Parkinson & Registrar, 1871; Walter et al., 1962). The neurovisceral integration model proposes a bi-directional cortical influences on autonomic functioning and integrates central nervous and autonomic systems (Smith et al., 2017; Thayer & Lane, 2000). The main affector and effector of the parasympathetic nervous system is the vagal nerve. Electrical vagal nerve stimulation seems to improve cognitive functioning, in particular memory consolidation and recognition (Clark et al., 1999; Ghacibeh et al., 2006; Vonck et al., 2014). Interestingly, also behavioural interventions such as slow breathing, associated with vagal nerve increased activity, are able to modulate cortical alpha rhythm (Hsu et al., 2020; Maric et al., 2020). Cardiovascular desynchronization, in turn, may have detrimental impact on cognition (Ogoh & Tarumi, 2019).

Evidence for cognitive improvements after HBOT in different neurological disorders accumulates (Boussi-Gross et al., 2015; Hardy et al., 2002; Rossignol et al., 2007; Tapeantong & Poungvarin, 2009). Nevertheless, there is still an ongoing debate regarding the efficacy of HBOT in individuals with traumatic brain injury (TBI), stroke or other neurological conditions. Although it is widely agreed, and confirmed by the majority of studies, that HBOT leads to significant improvements in cognition, the debate is mostly related to a control group issue and minimal effective dosages (the minimal pressure that does not have any physiological effect on the CNS) (Churchill et al., 2013; Efrati & Ben-Jacob, 2014a, b; Golden et al., 2006; Harch et al., 2007; Wolf et al., 2012). There are several neurological conditions where HBOT has been reported to be useful, that is, CO poisoning, TBI and post-concussion syndrome (PCS), stroke and neurodegenerative disorders.

Existing intensive functional therapy and neuropsychological rehabilitation programmes are considered essential to minimise cognitive and physical sequelae associated with brain damage in various neurological states. Neuropsychological rehabilitation refers to helping cognitively impaired individuals to totally or partially restore normal functioning, compensate cognitive, emotional, psychosocial and behavioural deficits, and improve the quality of life. However, considering the heterogeneity of physical, cognitive, behavioural, and psychosocial symptoms in various neurological states, these programmes are often only partially successful. Those programs should be improved and modified in accordance with the better understanding of cognitive processes and brain structures via newest neuroimaging techniques and medical development. It is well known that neuroplasticity and synaptic reorganization have a decisive role in neuropsychological rehabilitation effects (Sohlberg & Mateer, 2001). Some of the mechanisms involved in both processes have essential implications for rehabilitation, such as diaschisis, functional reorganization, or modification of synaptic connectivity among others (Sohlberg & Mateer, 2001). Thus, alternative approaches dedicated to the metabolic recovery of cerebral tissues that stimulates synaptic and functional plasticity such as HBOT need to be further explored in order to improve the neuropsychological outcome of a patient.

Finally, there are some adverse events and risks associated with HBOT. They are related to changes in atmospheric pressure, hyperbaric oxygen exposure and psychological factors (i.e. confinement anxiety). Middle ear barotrauma is the most common HBOT-related complication with incidence rates ranging between authors from 2 to 82%. In one of the largest retrospective study of HBOT safety the incident risk of middle era barotrauma was 9.2% of patients, which gives 410 events per 100,000 sessions. The safety of hyperbaric oxygen treatment was assessed in 2,334 patients (Hadanny et al., 2016b). To minimise this risk, patients are provided with instructions describing ear clearing techniques like swallowing, chewing and modified Valsalva manoeuvre. In intubated and unconscious patients myringotomy is the procedure of choice before the HBOT. Oxygen itself is a drug and there is always a risk of adverse effects based on biochemical reactions. CNS oxygen toxicity presented with the temporarily loss of consciousness and general seizures is quite infrequent event. It occurs with a rate of only 1.59 events per 100,000 sessions, mostly at pressures 2.4 ATA and above (Hadanny et al., 2016a). Seizures during hyperbaric oxygen therapy were assessed in retrospective analysis of 62,614 treatment sessions. This is one of the reasons why in case of using HBOT in emergency indications (i.e. diving accidents, CO intoxication, compartment syndrome, etc.) when high partial pressure of oxygen is used, use of multiplace chambers with direct supervision of medical attendant inside the chamber is preferred. If such event occurs, the internal attendant immediately removes the oxygen mask from the patient and switches him or her to air breathing. Lungs oxygen toxicity is cumulative, but can be avoided by using minimum inspired fractions of oxygen between HBOT sessions. In some patients with pre-existing cardiac problems, there is a risk of hypotension after HBOT due to hypovolemia evoked by vanishing vasoconstrictive effect. Hypovolemia risk can be mitigated by careful volume assessment and, if needed, volume loading before the end of oxygen exposure. Eyes oxygen toxicity, manifesting as myopia, may occur in patients receiving HBOT over several weeks. Myopia is usually spontaneously reversible within a short period of time. Gas embolism and pulmonary barotrauma, a well-known phenomena in divers, during decompression in hyperbaric chambers are very rare (Mathieu, 2006b; Oriani et al., 1996). In conclusion of the adverse effects, the HBOT is generally considered as a low risk medical procedure when conducted by the well trained hyperbaric personnel in carefully assessed patients.

## Methodology

PRISMA guidelines were used for the reporting of this systematic review (Moher et al., 2009).

### Data Sources

Electronic database Pubmed was searched, from 1946 to 2020, for relevant studies; last search was performed on 10 May 2020. The reference lists of included studies were hand-searched for additional references.

Separate search for four medical indications for HBOT (CO intoxication, post-concussion syndrome, stroke, ageing and neurodegenerative diseases) was made. The following search terms were used:

- CO intoxication Search: ((hyperbaric oxygen therapy) AND (carbon monoxide)) AND (cognition) ("hyperbaric oxygenation"[MeSH Terms] OR ("hyperbaric"[All Fields] AND "oxygenation"[All Fields]) OR "hyperbaric oxygenation"[All Fields] OR ("hyperbaric"[All Fields] AND "oxygen"[All Fields] AND "therapy"[All Fields]) OR "hyperbaric oxygen therapy"[All Fields]) AND ("carbon monoxide"[MeSH Terms] OR ("carbon"[All Fields] AND "monoxide"[All Fields]) OR "carbon monoxide"[All Fields]) AND ("cognition"[MeSH Terms] OR "cognition"[All Fields] OR "cognitions"[All Fields] OR "cognitive"[All Fields] OR "cognitively"[All Fields] OR "cognitives"[All Fields])

b) Traumatic brain injury Search: ((hyperbaric oxygen therapy) AND (concussion)) AND (cognition) Sort by: Most Recent ("hyperbaric oxygenation"[MeSH Terms] OR ("hyperbaric"[All Fields] AND "oxygenation"[All Fields]) OR "hyperbaric oxygenation"[All Fields] OR ("hyperbaric"[All Fields] AND "oxygen"[All Fields] AND "therapy"[All Fields]) OR "hyperbaric oxygen therapy"[All Fields]) AND ("brain concussion"[MeSH Terms] OR ("brain"[All Fields] AND "concussion"[All Fields]) OR "brain concussion"[All Fields] OR "concussion"[All Fields] OR "concussions"[All Fields] OR "concussed"[All Fields] OR "concussive"[All Fields]) AND ("cognition"[MeSH Terms] OR "cognition"[All Fields] OR "cognitions"[All Fields] OR "cognitive"[All Fields] OR "cognitively"[All Fields] OR "cognitives"[All Fields]) and Search: ((hyperbaric oxygen therapy) AND (traumatic brain injury)) AND (cognition) ("hyperbaric oxygenation"[MeSH Terms] OR ("hyperbaric"[All Fields] AND "oxygenation"[All Fields]) OR "hyperbaric oxygenation"[All Fields] OR ("hyperbaric"[All Fields] AND "oxygen"[All Fields] AND "therapy"[All Fields]) OR "hyperbaric oxygen therapy"[All Fields]) AND ("brain injuries, traumatic"[MeSH Terms] OR ("brain"[All Fields] AND "injuries"[All Fields] AND "traumatic"[All Fields]) OR "traumatic brain injuries"[All Fields] OR ("traumatic"[All Fields] AND "brain"[All Fields] AND "injury"[All Fields]) OR "traumatic brain injury"[All Fields]) AND ("cognition"[MeSH Terms] OR "cognition"[All Fields] OR "cognitions"[All Fields] OR "cognitive"[All Fields] OR "cognitively"[All Fields] OR "cognitives"[All Fields])
c) Stroke Search: ((hyperbaric oxygen therapy) AND (stroke)) AND (cognition) ("hyperbaric oxygenation"[MeSH Terms] OR ("hyperbaric"[All Fields] AND "oxygenation"[All Fields]) OR "hyperbaric oxygenation"[All Fields] OR ("hyperbaric"[All Fields] AND "oxygen"[All Fields] AND "therapy"[All Fields]) OR "hyperbaric oxygen therapy"[All Fields]) AND ("stroke"[MeSH Terms] OR "stroke"[All Fields] OR "strokes"[All Fields] OR "stroke s"[All Fields]) AND ("cognition"[MeSH Terms] OR "cognition"[All Fields] OR "cognitions"[All Fields] OR "cognitive"[All Fields] OR "cognitively"[All Fields] OR "cognitives"[All Fields]) and Search: ((hyperbaric oxygen therapy) AND (cognition)) AND (ischemia) ("hyperbaric oxygenation"[MeSH Terms] OR ("hyperbaric"[All Fields] AND "oxygenation"[All Fields]) OR "hyperbaric oxygenation"[All Fields] OR ("hyperbaric"[All Fields] AND "oxygen"[All Fields] AND "therapy"[All Fields]) OR "hyperbaric oxygen therapy"[All Fields]) AND ("cognition"[MeSH Terms] OR "cognition"[All Fields] OR "cognitions"[All Fields] OR "cognitive"[All Fields] OR "cognitively"[All Fields] OR "cognitives"[All Fields]) AND ("ischaemia"[All Fields] OR "ischemia"[MeSH Terms] OR "ischemia"[All Fields] OR "ischaemias"[All Fields] OR "ischemias"[All Fields])
d) Ageing and neurodegenerative disorders Search: ((hyperbaric oxygen therapy) AND (neurodegenerative disease)) AND (cognition) ("hyperbaric oxygenation"[MeSH Terms] OR ("hyperbaric"[All Fields] AND "oxygenation"[All Fields]) OR "hyperbaric oxygenation"[All Fields] OR ("hyperbaric"[All Fields] AND "oxygen"[All Fields] AND "therapy"[All Fields]) OR "hyperbaric oxygen therapy"[All Fields]) AND ("neurodegenerative diseases"[MeSH Terms] OR ("neurodegenerative"[All Fields] AND "diseases"[All Fields]) OR "neurodegenerative diseases"[All Fields] OR ("neurodegenerative"[All Fields] AND "disease"[All Fields]) OR "neurodegenerative disease"[All Fields]) AND ("cognition"[MeSH Terms] OR "cognition"[All Fields] OR "cognitions"[All Fields] OR "cognitive"[All Fields] OR "cognitively"[All Fields] OR "cognitives"[All Fields]) and Search: (hyperbaric oxygen therapy) AND (alzheimer disease) ("hyperbaric oxygenation"[MeSH Terms] OR ("hyperbaric"[All Fields] AND "oxygenation"[All Fields]) OR "hyperbaric oxygenation"[All Fields] OR ("hyperbaric"[All Fields] AND "oxygen"[All Fields] AND "therapy"[All Fields]) OR "hyperbaric oxygen therapy"[All Fields]) AND ("alzheimer disease"[MeSH Terms] OR ("alzheimer"[All Fields] AND "disease"[All Fields]) OR "alzheimer disease"[All Fields]) and Search: ((hyperbaric oxygen therapy) AND (neurodegeneration)) AND (cognition) Sort by: Most Recent ("hyperbaric oxygenation"[MeSH Terms] OR ("hyperbaric"[All Fields] AND "oxygenation"[All Fields]) OR "hyperbaric oxygenation"[All Fields] OR ("hyperbaric"[All Fields] AND "oxygen"[All Fields] AND "therapy"[All Fields]) OR "hyperbaric oxygen therapy"[All Fields]) AND ("nerve degeneration"[MeSH Terms] OR ("nerve"[All Fields] AND "degeneration"[All Fields]) OR "nerve degeneration"[All Fields] OR "neurodegeneration"[All Fields] OR "neurodegenerating"[All Fields] OR "neurodegenerations"[All Fields]) AND ("cognition"[MeSH Terms] OR "cognition"[All Fields] OR "cognitions"[All Fields] OR "cognitive"[All Fields] OR "cognitively"[All Fields] OR "cognitives"[All Fields]) and Search: ((hyperbaric oxygen therapy) AND (ageing)) AND (cognition) Sort by: Most Recent ("hyperbaric oxygenation"[MeSH Terms] OR ("hyperbaric"[All Fields] AND "oxygenation"[All Fields]) OR "hyperbaric oxygenation"[All Fields] OR ("hyperbaric"[All Fields] AND "oxygen"[All Fields] AND "therapy"[All Fields]) OR "hyperbaric oxygen therapy"[All Fields]) AND ("aging"[MeSH Terms] OR "aging"[All Fields] OR "ageing"[All Fields]) AND ("cognition"[MeSH Terms] OR "cognition"[All Fields] OR "cognitions"[All Fields] OR "cognitive"[All Fields] OR "cognitively"[All Fields] OR "cognitives"[All Fields])

### Study Selection and Eligibility Criteria

**Fig. 1:**
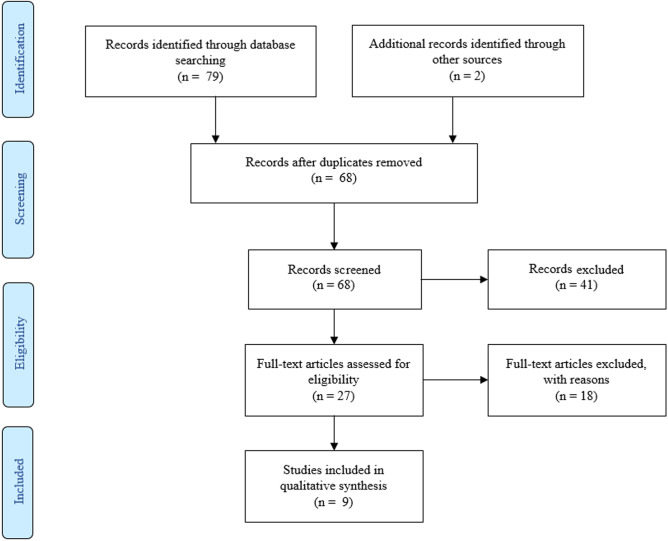
PRISMA Flow Diagram for Included Studies for HBOT usage in cognitive dysfunction after CO intoxication

**Fig. 2:**
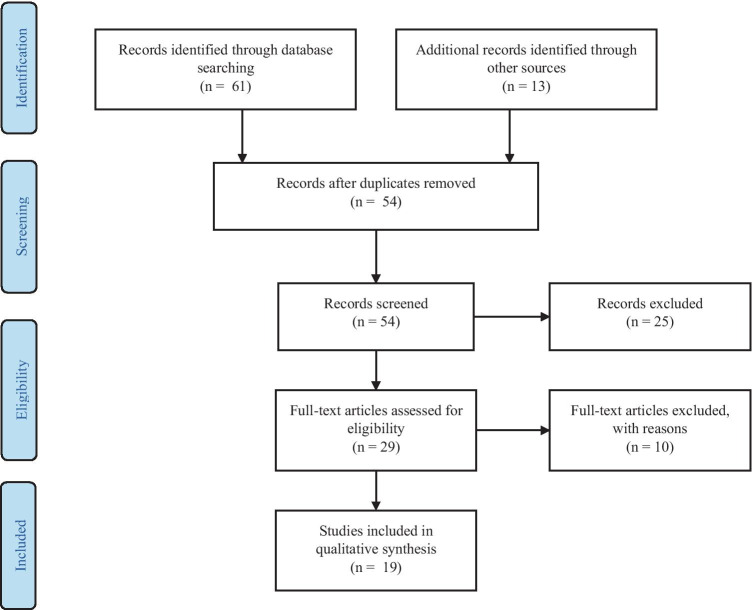
PRISMA Flow Diagram for Included Studies for usage of HBOT in TBI-related cognition disorders

**Fig. 3:**
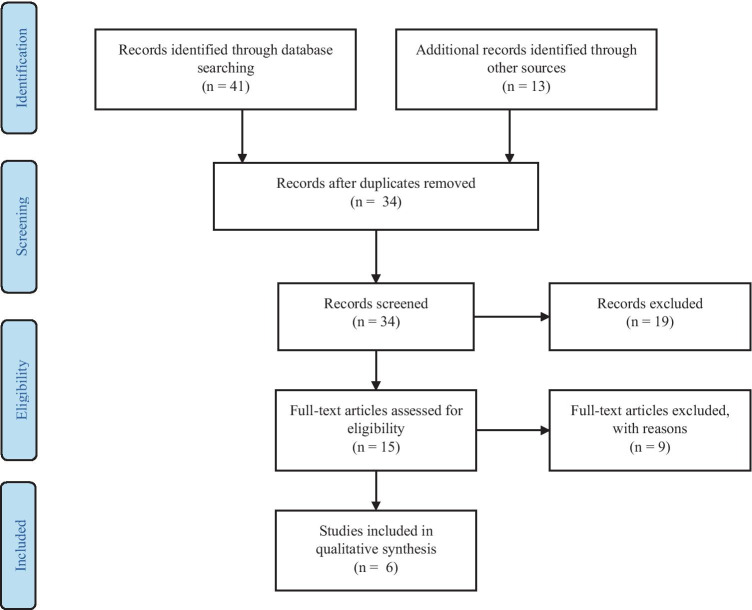
PRISMA Flow Diagram for Included Studies for HBOT usage in postroke cognitive disturbances

**Fig. 4:**
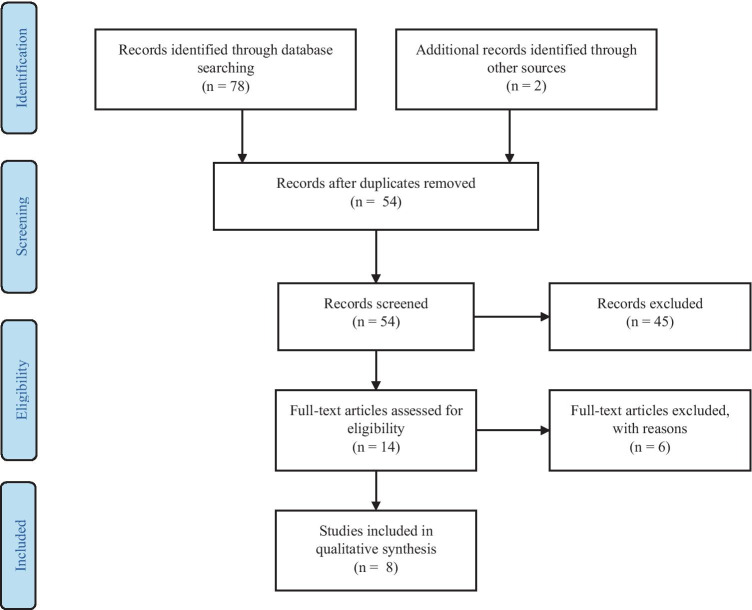
PRISMA Flow Diagram for Included Studies for HBOT in cognitive ageing and neurodegenerative disorders

A two-step approach was used to select articles. Firstly, titles and abstracts of all search results were screened for the following characteristics (1) original article published in English, (2) case studies were excluded. Secondly, full-text articles were obtained from the selected studies and were reviewed on the following inclusion criteria (1) performed cognitive processes assessment (2) performed HBO therapy with described HBOT protocol. Two neuropsychologists independently reviewed titles, abstracts, full texts and extracted data. The review process is summarized in Figs. 1, 2, 3 and 4. Results from the systematic literature search are provided separately in each indication section of the manuscript.

## Results and Discussion

### HBOT and Cognitive Dysfunctions After CO Intoxication

Exposure to CO, which excludes oxygen from tissues through the formation of a stable complex carboxyhaemoglobin (COHb), can damage various body systems – including the CNS. The acute symptoms reported by patients are: headache, weakness or lethargy, dizziness, nausea, shortness of breath, chest pain, visual changes and muscle cramping. Moreover, up to 45% have cognitive problems with memory, attention and concentration (Weaver et al., 2002). Patients in more severe states (with a loss of consciousness or with COHb levels greater than 25%) frequently show cognitive dysfunction lasting 1 month or longer (Goldstein, 2008; Weaver et al., 2002). Multiple mechanisms are involved in both acute and delayed CO toxicity, including neuroinflammation, apoptosis and brain lipid peroxidation. All of them are ameliorated by HBOT (reviewed by Mannaioni et al.,  2006).

Most patients with CO poisoning can recover from the acute phase. However, around 1–30% of patients develop delayed neuropsychological sequelae 2–6 weeks after recovery from the acute phase (Choi, 1983; Weaver, 2009; Weaver et al., 2007). Therefore, treatment for CO poisoning during the acute phase aims to address immediate threats to life as well as prevent delayed and sometimes permanent neuropsychological morbidity (Liao et al., 2019; Mathieu et al., 1985).

Gale and Hopkins (2004) recruited 20 patients with CO poisoning and assessed neuropsychological functioning. They showed a correlation between impairment of verbal memory and visual memory and reduced hippocampal volume in patients. Cognitive disturbances were present even 6 months or longer after CO poisoning. Chang et al. (2010) described similar findings about persistent neuropsychological deficits (verbal episodic memory, visual memory and visual-spatial ability) in 9 patients with CO exposure: these issues were still present after 3 and 10 months. Moreover T2-weighted brain magnetic resonance imaging of patients with delayed neuropsychiatric sequelae after CO poisoning shows damage to the hippocampus (Bruno et al., 1993; Henke et al., 1999) and white-matter lesion in the frontal lobe and periventricular area (Mundy et al., 2013; Park & Kim, 2014). Those regions are involved in reference memory and working memory, respectively (Burges et al., 2002; Prior et al., 1997).

The standard treatment for CO poisoning includes administration of 100% oxygen and general supportive care. Administration of supplemental oxygen increases the dissolved oxygen content, hastens the elimination of COHb in the blood and decreases cerebral oedema (Goldstein, 2008). HBOT is often recommended for patients with acute CO poisoning, especially if they have lost consciousness or have severe poisoning symptoms (Mathieu et al., 2017; Stoller, 2007).

There are also few animal studies in this field. Liu et al. (2016) sought whether neurogenesis is the target for HBOT to abrogate the delayed neuropsychological sequelae after CO poisoning. HBOT (2.5 ATA with 100% oxygen for 60 min) was conducted on rats during the first 7 days after CO poisoning. Their animal research suggests that early HBOT may ameliorate delayed neuropsychological disturbances after acute CO poisoning by promoting neurogenesis through upregulating Brain-Derived Neurotrophic Factor (BDNF) in the hippocampus. Furthermore, the same group of authors conducted study with HBO therapy combined with N-butylphthalide on 80 rats. HBOT was conducted on the same conditions as in previous study. Rats performed Morris Water Maze Task. Last part of the study included hippocampus microstructure assessment. Authors concluded that combined therapy can improve cognitive functioning through maintaining ultrastructural integrity of hippocampus, and thus may play a neuroprotective role in brain tissue in rats with CO poisoning. Animal studies examining effect of HBOT on cognition after carbon monoxide intoxication are presented in Table 1.

**Table 1:** Effect of HBOT in carbon monoxide poisoning on cognition in animals

Authors	Studied group	HBOT protocol	Cognitive measures	Results
(Liu et al., 2016)	10 rats after acute carbon monoxide poisoning	7 sessions for 60 min at 2.5 ATA	Eight arm maze test	Improvement in memory
(Bi et al., 2017)	80 rats after CO intoxication; divided in two groups HBOT and HBOT + N-butylphthalide	7 sessions for 60 min at 2.5 ATA	Morris Water Maze Task	Improvement in memory

*ATA* absolute atmosphere, *CO* carbon monoxide, *HBOT* hyperbaric oxygen therapy

Several studies have investigated the effect of HBOT with multiple neuropsychological methods. Scheinkestel et al. (1999) conducted a double-blind, randomised clinical trial to investigate the effects of HBOT by measuring cognitive functioning immediately after the therapy (daily treatments with 100% oxygen in a hyperbaric chamber – 60 min at 2.8 ATA for the HBOT group and at 1.0 ATA for the normobaric oxygen group – for three days) and 1 month later. They found no improvement in the patients’ states, and they reported a worse outcome in learning tests.

A similar methodology was used by Weaver et al. (2002); they examined 152 patients with CO poisoning with various neuropsychological tests after the first and third session and at 2 and 6 weeks, 6 months and 12 months after HBOT. All subjects underwent three protocol-directed sessions in monoplace hyperbaric chambers at intervals of 6 to 12 h. The first oxygen treatment session was initiated within 24 h after the end of the exposure to CO. During both hyperbaric-oxygen and normobaric-oxygen sessions, all intubated patients were mechanically ventilated with 100% oxygen. The patients in the HBOT group were exposed to 100% oxygen at 3 ATA and then 2 ATA during the first chamber session and then to 100% oxygen at 2 ATA during sessions 2 and 3. Subjects in the normobaric-oxygen group were exposed to air at 1 ATA for all three chamber sessions. After three sessions, there were improvement in a few neuropsychological tests (Digit Span, Trail Making, Digit-Symbol, Block Design and Story Recall), demonstrating increased attention, visuo-spatial and memory processes as well as processing speed. This avoided two of the limitations of the study by Scheinkestel et al. (1999), i.e. loss to follow up and cluster rather than patient level randomisation for group exposures. Thus, it was the first study that was not plagued with or accused of having methodological flaws. From this study, 44 individuals were subsequently enrolled to ancillary prospective *APOE* genotyping study. The authors found that HBOT reduced cognitive deficits after CO poisoning in the absence of the e4 allele and concluded that HBOT is a useful treatment for preventing cognitive sequalae after CO poisoning (Hopkins et al., 2007).

Delayed neuropsychiatric sequel is estimated to occur in 10–30% of victims of carbon monoxide poisoning, but the reported incidence varies widely (Sönmez et al., 2018). Its various symptoms mainly comprise cognitive impairment, parkinsonism, urinary and faecal incontinence, dementia and psychosis (Choi, 1983; Tapeantong & Poungvarin, 2009). Emergent HBOT within 24 h reduces the risk of cognitive sequelae after acute CO poisoning (Weaver et al., 2002).

Yeh et al. (2014) indicated that patients with delayed neuropsychiatric sequel after CO intoxication had poorer performance on general cognitive functioning, language skills, psychomotor speed, visual-spatial processes, logical and working memory and executive function compared to those with acute CO poisoning at 1 month. Compared with the acute neuropsychological sequel group, the group with delayed symptoms had more significant progress at 6-month follow-up after HBOT with regard to general cognitive function, psychomotor speed and visuo-spatial skills. However, the follow-up progress on language processes, logical memory and executive function tasks did not differ between the groups. These results support previous brain-image findings that patients with delayed neuropsychiatric sequel had abnormal deep white matter and frontal lobe regions (Ernst & Zibrak, 1998; Zagami et al., 1993). The authors suggested that future research should employ standardised and comprehensive neuropsychological tasks, as well as larger samples and matching brain-imaging investigations.

Lo et al. (2007) performed magnetic resonance, diffusion tensor imaging and Mini-Mental State Examination (MMSE) examination in 6 patients with delayed neuropsychiatric sequel immediately before and 3 months after HBOT (subjects underwent from 8 to 40 consecutive sessions, depending on the clinical response, with a pressure of 2.5 ATA for 120 min in each session) to obtain fractional anisotropy values and assess neuropsychological functioning. There was a significantly higher mean fractional anisotropy value in the control group compared with the patients both before and 3 months after HBOT. Notably, in the individuals with delayed symptoms of CO poisoning, the mean fractional anisotropy value 3 months after HBOT was also significantly higher than before HBOT. All of the patients regained full scores in the MMSE 3 months after the hyperbaric oxygen therapy.

Chang et al. (2010) examined 9 patients – with symptoms of delayed neuropsychiatric sequel – who received HBOT (at 2.5 ATA for 120 min five days per week during hospitalisation; 8–40 sessions). For all patients, cognitive symptoms significantly improved after the therapy, with significantly higher MMSE scores. However, white matter changes remained evident in the brain magnetic resonance scans.

Although HBOT has been applied clinically for the treatment of both acute and delayed phase of CO poisoning and HBOT reduces the neurological sequelae caused by the poison, the clinical benefits and the underlying mechanisms are still controversial (Annane et al., 2011; Birmingham & Hoffman, 2011; Camporesi, 2014; Hampson et al., 2013; Weaver et al., 2007). Nevertheless, growing evidence has indicated that HBOT can promote proliferation of endogenous neural stem cells and stimulate neurogenesis in the injured brain (Lee et al., 2013; Mu et al., 2011; Zádori et al., 2011).

Delayed encephalopathy after acute CO poisoning (DEACMP) is a very serious complications (Goldstein, 2008). DEACMP is a common cause of clinical neurological complications and may result in memory impairment, unresponsiveness, visceral autonomic nervous system dysfunction, Parkinson’s disease, cognitive dysfunction and behavioural disorders in patients. Currently, the pathogenesis of DEACMP remains elusive. Given that the CNS is the tissue that is most sensitive to oxygen, some researchers have sought primary factors that induce DEACMP in hypoxia caused by CO poisoning (Lee et al., 1988; Prockop & Chichkova, 2007). This theory does not explain all clinical manifestations and pathological changes present in DEACMP, especially the variety of symptoms that occur after the recovery of the carbonyl haemoglobin level. Other researchers have reported that delayed CO poisoning neuropathology is associated with an adaptive immunological response to chemically modified myelin basic protein (Thom et al., 2004). Xiang et al. (2017a, b), in their double-blind, randomised study involving 215 DEACMP patients (MMSE score ≤ 24, indicative of cognitive delay), found that both HBOT and the combined application of N-butylphthalide and HBOT (with 2.5 ATA for 80 min per day) significantly increased cognitive functioning (measured with MMSE) after short-term treatment. The efficacy of the combined treatment was greater than HBOT alone. After 8 weeks of treatment, almost half of the experimental group achieved significantly higher results compared to the control group. The researchers concluded that HBOT may increase the blood oxygen level to alleviate the hypoxic state in the brain of CO-poisoned patients. This phenomenon is beneficial to the functional recovery of damaged brain cells.

Moreover, the combined application of N-butylphthalide and HBOT may be a potential effective therapy in treating cognitive dysfunction for patients with DEACMP. Furthermore, the same authors conducted study on 120 DEACMP patients divided in two groups: HBOT alone and HBOT plus dexamethasone. Each patients received 20 hyperbaric oxygen therapy sessions at 2.5 ATA for 80 min per day. MMSE performed before and after 4 weeks of treatment showed cognitive improvement in both groups. Although authors concluded that the combined application of dexamethasone and HBO therapy should be considered as it yields better efficacy for patients with DEACMP (Xiang et al., 2017a, b). Efficacy of N-butylphthalide and dexamethasone combined with HBOT in patients with DEACMP was also studied in recent study by Zhang et al. (2020). Authors examined 171 DEACMP patients and divided in two groups: combined therapy and HBO alone. Cognitive state was assessed with MMSE and Montreal Cognitive Assessment (MoCA) scale before and 1 and 3 months and 1 year after the treatment. Their results indicate that combined therapy can significantly improve cognitive and motor functions of patients with DEACMP. Human studies examining effect of HBOT on cognition after carbon monoxide intoxication are presented in Table 2.

**Table 2:** Effect of HBOT in carbon monoxide poisoning on cognition in human

Authors	Studied group	HBOT protocol	Cognitive measures	Results
(Scheinkestel et al., 1999)	Patients referred to acute CO poisoning; 104 subjects received HBO therapy	3–6 days, 1 session per day, HBOT for 60 min at 2.8 ATA	Neuropsychological assessment performed after treatment; Simple reaction time, Choice reaction time, Digit span forward and backward, Rey Auditory Verbal Learning, MMSE	No improvement; worsening in memory tests
(Weaver et al., 2002)	Symptomatic acute carbon monoxide poisoning; 76 subjects	3 chamber sessions within 24 h; 3 HBO sessions; 1 day of HBOT performed at 3 then 2 ATA, day 2 and 3 at 2 ATA	Neuropsychological tests were administered immediately after, 2 and 6 weeks, 6, and 12 months after HBO; Digit Span, Trail Making Test, Digit-Symbol, Block Design and Story Recall	Increased attention, visuo-spatial, memory processes and processing speed
(Lo et al., 2007)	6 patients with delayed neuropsychiatric sequel caused by CO intoxication	8–40 HBOT sessions for 120 min at 2.5 ATA	MMSE performed immediately before and 3 months after the HBOT	Cognitive improvement (patients regained full scores in the MMSE)
(Chang et al., 2010a, b)	9 patients with delayed neuropsychiatric sequel after CO intoxication	8–40 sessions at 2.5 ATA for 120 min five days per week	MMSE performed before and after HBOT	Cognitive improvement (higher MMSE scores)
(Xiang et al., 2017b)	215 DEACMP patients; divided in two groups HBOT and HBOT N-butylphthalide	40 sessions at 2.5 ATA for 80 min per day; 5 sessions per week for 8 weeks	MMSE performed before and after HBOT	Cognitive improvement in both groups
(Xiang et al., 2017a)	120 patients with DEACMP; divided in two groups HBOT and HBOT + dexamethasone	20 HBOT sessions, 5 sessions per week at 2.5ATA for 80 min per day	MMSE performed before and after treatment	Cognitive improvement in both groups
(Zhang et al., 2020)	171 DEACMP patients; divided in two groups: HBOT and HBOT + N-butylphthalide + dexamethasone	20 HBOT sessions, 5 sessions per week at 2.5ATA for 80 min per day	MMSE and MoCA scale performed before and 1 and 3 months and 1 year after HBOT	Cognitive improvement in both groups

*ATA* absolute atmosphere, *CO* carbon monoxide, *HBOT* hyperbaric oxygen therapy, *MMSE* Mini-Mental State Examination, *MoCA* Montreal Cognitive Assessment, *DEACMP* delayed encephalopathy after acute CO poisoning

Current studies examining CO intoxication effects mostly agree that HBOT is promising therapy for improving patients cognition. Although research paradigm varies (e.g. therapy range from 3 to 40 HBOT sessions), thus comparing results should be made with caution. Furthermore, detailed neuropsychological assessment is rarely done. Cognitive screening methods (i.e. MMSE, MoCA) are not reliable and sensitive enough to detect subtle changes in cognitive functioning. Future studies should be designed to determine optimal combinations of the dose and timing of HBOT, and planned subgroup analyses should attempt to define which patients could benefit most from hospital transfer. These studies will hopefully provide the evidence needed to eliminate the remaining doubt about the effectiveness of HBOT. More aggressive and appropriate treatment options than are used today are needed. The correct diagnosis of the cognitive consequences of CO poisoning often occur quite a bit after the exposure. Thus, appropriate and timely treatment is even more problematic.

### HBOT and TBI-Related Cognition Disorders

TBI can be caused by several factors. An external physical force, rapid acceleration or deceleration of the head, bleeding within or around the brain, lack of sufficient oxygen to the brain or toxic substances passing through the blood–brain barrier. The damage caused by TBI can be focal (confined to one area of the brain) or diffuse (involving more than one area of the brain: Zhang et al., 2014). Symptoms of a TBI vary from mild and moderate to severe, depending on the extent of the damage to the brain. TBI can result in temporary or permanent impairment of cognitive, emotional or physical functioning state. Thus, it has become a significant concern in civilian and military populations (Chiu & LaPorte, 1993). TBI is linked with several pathological mechanisms, that is, diffuse shearing of axonal pathways and small blood vessels – which is also known as diffuse axonal injury (Medana & Esiri, 2003) – ischaemia, mild oedema and other biochemical and inflammatory processes, that culminate in impaired regenerative or healing processes resulting from increasing tissue hypoxia (Zasler et al., 2007). Furthermore, TBI can lead to PCS, a complex of symptoms such as headaches, dizziness, imbalance, vertigo, fatigue, changes in sleep pattern, neuropsychiatric symptoms (e.g., behavioural and mood changes, confusion) and cognitive impairments (in memory, attention, concentration and executive functions) (McCauley et al., 2000). Due to multiple pathological mechanisms, cognitive impairments are usually the predominant symptoms localised in multiple brain areas (Kushner, 1998; Levin, 1990; Sohlberg & Mateer, 2001a, b). The use of HBOT for brain injury is based on the hypothesis that injured or inactive neurons would benefit from increased blood flow and oxygen delivery, which would act to metabolically or electrically reactivate the cells (Deng, 2018; Francis & Baynosa, 2017; Neubauer & James, 1998).

In recent years, animal models (Chen et al., 2010; Efrati & Ben-Jacob, 2014a, b; Lin et al., 2012; Neubauer & James, 1998) and human studies (Barrett et al., 2004; Boussi-Gross et al., 2013; Golden et al., 2006; Harch et al., 2012; Shi et al., 2003; Tal et al., 2015; Wright et al., 2009) have shown that HBOT can improve PCS by targeting basic pathological processes (Hadanny & Efrati, 2016). The evidence about the effectiveness of HBOT for TBI is conflicting, and the case series and time series studies of HBOT for TBI patients had serious flaws. One of the most concerning issues is the optimal time window for HBOT, a factor that can determine its efficacy in TBI.

Animal model studies have demonstrated that HBOT has a positive effect on cognitive outcome (Kraitsy et al., 2014; Lin et al., 2012). Zhou et al. (2007) found that rats with brain injury treated with HBO (1 h at 1.5 ATA + 3hour normobaric oxygen) had significant improvement in cognitive recovery. HBO research using sham, NBO and HBO was used by Harch et al. (2007). Authors proved that 80 HBOT sessions performed twice a day for 90 min at 1.5 ATA can significantly improve spatial learning in TBI rats. They confirmed their results with showing increase in hippocampus vascular density after hyperbaric oxygen therapy. Wang et al. investigated the prolonged therapeutic time window of HBOT in animal models. Their study involved HBOT administration within 6 h after TBI. Their results showed decreased neuronal apoptosis and improved cognitive ability. Furthermore, they described that multiple HBOT sessions (3 ATA hourly for 3 or 5 days) could extend the therapeutic time window up to 48 h post-TBI (Wang et al., 2010). The authors also reported that a treatment initiated within 12 h after injury improved neurologic outcomes, compared with a longer window of 24 h. They concluded that 72 h after injury there is no significant improvement after single a HBOT session. However, if the first HBOT starts at 24 h after concussion and continues for 3 or 5 consecutive days, there are significant improvements in cognitive deficits. Their results suggest that the optimal HBOT paradigm for human studies may be a single treatment initiated within 24 h after the injury, followed by treatments for 5 consecutive days. Liu et al. (2013) investigated the effect and mechanism of HBOT on cognitive functioning in rats. Authors suggest that hyperbaric oxygen therapy significantly improves spatial learning and memory skills at rats with traumatic brain injury, and the potential mechanism behind those improvements is mediated by metabolic changes and nerve cell restoration in the hippocampus. Another research proving neuroprotective effect of HBOT after TBI was conducted by Baratz-Goldstein et al. (2017). They investigated impact of 4 consecutive days hyperbaric oxygen treatment on mice with traumatic brain injury (2 different time lines: 3 h after injury and 7 days post injury). They found that mice treated with hyperbaric oxygen showed significant improvement in learning abilities. Their results suggest neuroprotective effect of HBOT in TBI with short and long therapeutic window. Similar results (memory improvement) were described by Chen et al. (2014). Moreover they described therapeutic effect of hyperbaric oxygen on neuroinflammation, apoptosis and oedema after TBI.

The data from these pre-clinic and clinic studies indicate that HBOT is beneficial when it is applied early after an insult or injury. Application of HBOT within a therapeutic time window established in preclinical study is an important requirement to ensure treatment efficiency. Studies examining effect of HBOT on cognition in animal models of TBI are presented in Table 3.

**Table 3:** Effect of HBOT in traumatic brain injury on cognition in animals

Authors	Studied group	HBOT protocol	Cognitive measures	Results
(Zhou et al., 2007)	23 TBI rats	HBO for 1 h at 1.5 ATA	Morris water maze post injury days 11 to 15	Improvements in cognition
(Harch et al., 2007)	19 TBI rats	80 HBOT, twice a day, 7 days/week 1.5 ATA for 90 min	Morris water maze	Improvement in spatial learning
(Wang et al., 2010)	6 rats with TBI	3 ATA hourly for 3 or 5 days	Beam-balancing test; Prehensile traction test	Improved cognitive ability
(Liu et al., 2013)	20 rats with TBI	HBOT for 60 min daily at 2 ATA for 1 and 2 weeks	Morris water maze test immediately after TBI, 1 and 2 weeks of HBOT	Improvement in spatial learning and memory
(Baratz-Goldstein et al., 2017)	15 mice with TBI	HBOT session for 4 days for 60 min at 2 ATA	7 and 30 days after TBI; Elevated plus maze, Y-maze, Novel object recognition test	Improvement in learning abilities
(Chen et al., 2014)	9 TBI mice in each test	HBOT performed 3 h after injury for 60 min at 2 ATA for 5 days	Morris water maze (postinjury days 14,15,16 and 17), beam walk task (1,3,7 and 14 days post injury)	Improved motor skills and spatial learning
(Zhou et al., 2007)	23 TBI rats	HBO for 1 h at 1.5 ATA	Morris water maze post injury days 11 to 15	Improvements in cognition

*ATA* absolute atmosphere, *HBOT* hyperbaric oxygen therapy, *TBI* Traumatic Brain Injury

Cifu et al. (2014) conducted a randomised, blinded clinical study on subjects with persistent post-concussion symptoms. In their study – a randomised, controlled trial including 50 military service members suffering mild TBI between 3 and 71 months before HBOT. HBOT was assessed at 2.0 ATA. All subjects were randomly assigned to one of three groups, breathing 10.5%, 75% or 100% oxygen to mimic normal air at 1.0 ATA, 100% oxygen at 1.5 ATA or 100% oxygen at 2.0 ATA, respectively. HBOT at either 1.5 or 2.0 ATA equivalent had no effect on post-concussion symptoms after mild TBI when compared with sham compression. However, the HBOT effect on cognitive functions was assessed with the self-administered Rivermead Post-Concussion Symptoms Questionnaire (RPQ), which is known to display several flaws in implementation and in its ability to accurately reflect test-taker experience. Moreover, interpretation and accuracy of the RPQ can vary widely due to self-administration and the various confounding variables involved. Indeed, it is sensitive to subjective patient memory, social desirability, stress and other covariates such as personality factors and willingness to reveal problems, as are the two other methods. Relying completely on the self-administration assessments is a weakness of this study (Potter et al., 2006).

Furthermore, Walker et al. (2014) conducted a randomised, double-blind and sham-control feasibility trial comparing pretreatment and posttreatment conducted in 60 male active-duty marines with combat-related mild TBI. Subjects with PCS that had persisted for 3 to 36 months were randomised to one of three groups receiving preassigned oxygen fractions (10.5%, 75% or 100%) at 2.0 ATA. This design led to groups with an oxygen exposure equivalent to breathing surface air, 100% oxygen at 1.5 ATA, and 100% oxygen at 2.0 ATA, respectively. Each subject received 40 hyperbaric chamber sessions of 60 min each for 10 weeks. Multiple neuropsychological tests of cognitive performance were collected preintervention and 1-week postintervention. There were no significant changes in cognitive functioning between groups and pre and post HBOT. The authors concluded that HBOT is not useful to treat cognitive, balance or fine motor deficits associated with mild TBI and PCS.

Harch et al. (2012) performed an uncontrolled HBOT trial of 16 participants with PCS after blast exposure during military service. There was an improvement in both cognitive and psychomotor characteristics. The authors reported significant improvement one week after HBOT (40 at 1.5 ATA, 5 days per week for 60 min each session) in full-scale intelligence quotient (IQ), delayed and working memory, executive functions and attention. In a randomised trial, Wolf et al. (2012) studied participants with chronic PCS. Their results demonstrated no efficacy in cognitive impairment treatment with HBOT at an exposure pressure of 2.4 ATA for 90 min given once daily for 30 treatments compared to 1.3 ATA air exposure. However, both groups (the control group received treatment with room air at 1.3 ATA) improved beyond what would be expected more than 6 months after mild TBI. In subsequent study, they received similar results – no significant difference was observed between air (1.3 ATA) and HBO (2.4 ATA) although both groups shoved improvement in cognition (without statistical significance). The Wolf et al. (2012) studies were widely criticised for using 1.3 ATA in control group (Mychaskiw & Stephens, 2013; Weaver et al., 2013). Actually, it should be recognized that 1.3 ATA air and 2.4 ATA oxygen may represent low and high edges of the oxygen dose–response curve. Hyperbaric physiology indicates that relatively subtle changes in tissue partial oxygen pressure may exert a significant therapeutic effect (Mychaskiw & Stephens, 2013; Weaver et al., 2013).

Boussi-Gross et al. (2013) presented a prospective, randomised and controlled crossover study of the effect of HBOT with 100% oxygen at 1.5 ATA (5 days per week, 60 min each) on mild TBI patients at late chronic stage. The authors randomly divided the subjects into treated or crossover groups. The patients in the treated group were evaluated before and after 40 HBOT sessions. Subjects in the control group were evaluated at three times, baseline, and after 2 and 4 months. Neuropsychological examination included assessment of information processing, attention, memory and executive functions. All subjects underwent single photon emission computed tomography examination. HBOT induced neuroplasticity and significant brain function improvement in mild TBI patients with prolonged PCS (at a late chronic stage, years after brain injury). There was also improvement in all assessed cognitive functions. Moreover, changes in single photon emission computed tomography images after treatment indicate that HBOT reactivates neuronal activity in stunned areas that seem normal under computed tomography and magnetic resonance imaging.

Tal et al. (2015), in their study using perfusion magnetic resonance imaging, proved that HBOT can significantly increase cerebral blood flow and cerebral blood volume following 50–70 daily hyperbaric sessions, 5 days a week (each session consisted of 60 min of exposure to 100% oxygen at 1.5 ATA). There was also significant cognitive improvement in patients post TBI. The mean time from the acute injury was 10.3 ± 3.2 years. The most prominent improvements were seen in information processing speed, visual spatial processing and motor skills indices. The increased perfusion to the dysfunctional tissue, and the significant cognitive improvement, suggest that impaired tissue perfusion may serve as a rate limiting factor for regeneration and neuroplasticity even years after the acute injury. The authors concluded that that appropriate biological trigger can induce neuroplasticity months to years after the acute injury.

In a subsequent, study Tal et al. (2017) evaluated diffusion tensor imaging changes before and after HBOT of prolonged PCS. HBOT was initiated 6 months to 27 years (10.3 ± 3.2 years) from injury for 60 daily hyperbaric sessions, 5 days per week with sessions consisting of 90 min of exposure to 100% oxygen at 2 ATA. The authors found an increase in fractional anisotropy and a decrease in mean diffusivity after HBOT, together with cognitive function improvement of patients in the late chronic stage of TBI. Their results suggest that HBOT can induce brain microstructure recovery with significant improvement in memory, executive functions and information processing speed. They concluded that HBOT can improve the integrity of brain fibres, a phenomenon that correlates with improved cognitive functioning. It should be kept in mind, however, that quantitative validation of diffusion tensor imaging pathologic metrics remains very limited (Winklewski et al., 2018).

Churchill et al. (Churchill et al., 2016) examined effect of HBOT on information processing speed after mTBI. In their study, investigators measured speed of processing at baseline, 6 and 13 weeks in military personnel with mTBI. Subjects were randomized to 40 sessions of air (1.2 ATA) or HBOT (1.5 ATA). They found no significant changes in reaction time between HBO or air.

Furthermore Shandley and colleagues found that HBOT (90 min at 2.4 ATA) correlates with stem cell mobilization as well as increased neuropsychological performance comparing to 90 min air at 1.3 ATA. Authors suggest that stem cell mobilization may be required for cognitive improvement in TBI population.

Hadanny et al. (2018) demonstrated the neurotherapeutic effects of HBOT for chronic TBI. The study included 154 subjects (42.7 ± 14.6 years) with documented TBI 0.3–33 years (mean 4.6 ± 5.8, median 2.75 years) prior to HBOT. The HBOT protocol comprised 40–70 daily hyperbaric sessions, 5 days a week. Each session involved exposure to 100% oxygen at 1.5/2 ATA. The authors confirmed that HBOT induced significant improvement in memory, executive functions, information processing speed and global cognitive scores.

Weaver et al. (2012) proposed the design of randomised study to evaluate the efficacy and utility of HBOT for American combatants with PCS. Based on this design authors conducted clinical trial. They examined executive functions, processing speed, memory, and learning of 71 military service members with mTBI (35 with PTSD) who were randomly administered to HBO or air (40 HBO sessions at 1.5 ATA vs air at 1.2 ATA for 60 min). Authors found that after 13 weeks HBOT improved post-concussive and PTSD symptoms, cognitive processing speed, sleep quality, and balance function, especially in individuals with PTSD. Although changes did not persist beyond six months (Weaver et al., 2018). Research on effect of HBOT on cognition in traumatic brain disorders in humans are presented in Table 4.

**Table 4:** Effect of HBOT in traumatic brain injury on cognition in human

Authors	Studied group	HBOT protocol	Cognitive measures	Results
(Cifu et al., 2014)	61 male Marines with mTBI and PCS	40 HBOT sessions, 1 daily, sham or 100% oxygen at 1.5 ATA or 100% oxygen at 2 ATA	Rivermead Post-Concussion Questionnaire – 16	No significant changes
(Walker et al., 2014)	40 male Marines with mTBI	40 HBOT for 60 min at 1.5ATA or 2ATA	Wechsler Test of Adult Reading, Conners Continuous Performance Test-II, Paced Auditory Serial Addition Test, Halsted-Reitan Trail Making Test A & B, Stroop test, California Verbal Learning Test-II, Wechsler Adult Intelligence Scale III select items: digit symbol coding, digit span, letter-number sequencing, symbol search, and arithmetic, Delis-Kaplan Executive Function Systems version of the Controlled Oral Word Association Test single-letter and category items, Benton Visual Memory Test–Revised, Test of Memory Malingering	No significant changes
(Harch et al., 2012)	16 male TBI patients	40 sessions of HBOT at 1.5 ATA/60 min in twice a day/30 days	Green Word Memory Test, Effort Wechsler Test of Adult Reading, Rivermead Post-Concussion Symptom Questionnaire, Wechsler Adult Intelligence Scale-IV, Test of Variables of Attention, Stroop Test, Finger Tapping Test, Grooved Pegboard, Wechsler Memory Scale-IV, Rivermead Paragraph Memory	Improvement in delayed memory, working memory, Stroop test, Rivermead Post-Concussion Questionnaire – 16
(Wolf et al., 2012)	25 TBI patients	30 HBOT at 2.4 ATA and 1.3 ATA air sessions for 90 min	Immediate Post-Concussion Assessment and Cognitive Testing, Post-Traumatic Disorder Check List	No effect of HBOT
(Wolf et al., 2015)	25 TBI patients	30 HBOT at 2.4 ATA and 1.3 ATA air sessions for 90 min	Immediate Post-Concussion Assessment and Cognitive Testing, Post-Traumatic Disorder Check List	No effect of HBOT
(Boussi-Gross et al., 2013)	56 male TBI patients with PCS	40 HBOT sessions (5 days/week), 60 min with 100% oxygen at 1.5 ATA	Mindstreams: Verbal memory, Non-vernal memory, Go-No-Go test, Stroop test, Staged information processing test, Catch game	Significant improvement in all cognitive measures
(Tal et al., 2015)	10 TBI patients	60 HBOT sessions; 5/week at 1.5 ATA for 60 min	NeuroTrax—Verbal Memory, Non-Verbal Memory, Go-No-Go, Problem Solving, Stroop test, Finger Tapping, Catch Game, Staged Information Processing Speed, Verbal Function, and Visual Spatial Processing	Improvement in information processing speed, visual spatial processing and motor skills
(Tal et al., 2017)	15 TBI patients	60 daily HBOT sessions, 5/week for 90 min at 2 ATA	NeuroTrax—Verbal Memory, Non-Verbal Memory, Go-No-Go, Problem Solving, Stroop test, Finger Tapping, Catch Game, Staged Information Processing Speed, Verbal Function, and Visual Spatial Processing	Improvement in the memory, executive functions, information processing, speed and global cognitive scores
(Churchill et al., 2016)	23 TBI patients	40 HBOT sessions at 1.5 ATA for 60 min	Automated Neuropsychological Assessment Metrics: Simple Reaction Time and Procedural Reaction Time subtests	No significant changes
(Shandley et al., 2017)	15 TBI patients	30 HBOT sessions at 2.4 ATA for 90 min	Immediate Post-Concussion Assessment and Cognitive Testing, BrainCheckers	Increased cognitive performance
(Amir Hadanny et al., 2018)	154 TBI patients	40–70 daily HBOT sessions, 5/ week for 60/90 min at 1.5/2 ATA	NeuroTrax—Verbal Memory, Non-Verbal Memory, Go-No-Go, Problem Solving, Stroop test, Finger Tapping, Catch Game, Staged Information Processing Speed, Verbal Function, and Visual Spatial Processing	Improvement in memory and attention
(Weaver et al., 2018)	36 TBI patients	40 HBO sessions at 1.5 ATA for 60 min	Automated Neuropsychological Assessment Metrics, California Verbal Learning Test – II, Brief Visuospatial Memory Test – Revised, Test of Memory Malingering, Wechsler Adult Intelligence Scale – IV, digit span and processing speed Wechsler Test of Adult Reading, Stroop color and word test, Controlled oral word association test, Trail making test – parts A and B, Grooved pegboard, Trait Anger Expression Inventory	Improvement in post-concussive and PTSD symptoms, cognitive processing speed, sleep quality

*ATA* absolute atmosphere, *HBOT* hyperbaric oxygen therapy, *PCS* Post-Concussion Syndrome

Currently, the results of HBOT in clinical TBI trials are controversial, and the efficiency of HBOT in TBI has not been well established. First, the optimal time window for HBOT administration must be determined to ensure its efficacy in treating TBI. Second, objective and precise neuropsychological assessment methods are another challenge in the evaluation of the efficacy of HBOT in TBI patients. Third, heterogeneity in patients and HBOT paradigms (pressure, frequency, length of treatment course) partly affect or determine the outcome. There have been variations in patients’ age and in the severity and nature of the injury in the studies. Future trials of HBOT for PCS should consider measuring outcomes with standardised neuropsychological methods and at longer intervals postintervention or in combination with rehabilitation therapy to determine potential delayed or priming effects.

### HBOT and Poststroke Cognitive Disturbances

Stroke is a result of a blocked artery or a ruptured blood vessel. It leads to an interruption in cell homeostasis and symptoms such as loss of speech and loss of motor function. It is a major cause of disability and mortality among adults, with long-term impairments in the physical, emotional and cognitive state of survivors (Robinson, 2006). Neuropsychological disturbances after stroke are very common; they involve multiple cognitive deficits that lead to a decline in everyday functioning and in social functioning (Godefroy & Bogousslavsky, 2007). The main therapeutic targets are the regions surrounding the focal site of injury where the tissue is at high risk of disruption but not irreparably damaged; thus, there is still the potential to salvage these neurons (Baron, 2001; Lo et al., 2003; Singhal, 2007). Cell death and reduced neuronal activity caused by an ischaemic event can lead to excitotoxicity, oxidative stress, inflammation and apoptosis, all of which are pathways where hypoxia plays a key role (Lo et al., 2003). Therefore, increased oxygenation has been considered as a potential treatment for stroke; this treatment may lead to tissue repair and the generation of new synaptic connections (Golden et al., 2002; Neubauer & James, 1998). Therapy and neuropsychological rehabilitation programmes are valuable for improving cognition at early stages, but they usually provide only partial recovery from symptoms. To date, there is no efficient neuropsychological rehabilitation programs available for late chronic stages (Rajeswaran, 2013; Ricker & Callahan, 2000).

Current concepts of the pathophysiology of stroke provide a rationale for using HBOT in its management. Conventional methods of stroke treatment and their functional consequences are not satisfactory, and the outcomes remain controversial. There are only a few experimental animal studies and uncontrolled human trials that have shown the effectiveness and safety of HBOT after stroke. Clinical observations and basic research data suggest that HBOT may be a useful and effective treatment option in the management of acute stroke, but more studies are needed to clarify its clinical utility (Sánchez, 2013). The use of HBOT as a treatment following stroke was first raised 50–60 years ago (Hart & Strauss, 2003; Hart & Thompson, 1971). Despite decades of interest, studies that have investigated the effects of HBOT following a stroke have produced mixed results (Bennett et al., 2014; Freiberger et al., 2016; Helms et al., 2005). Rehabilitation of stroke patients is one aspect that should be planned during the first few months following stroke. Thus, long-term follow-up studies are required to determine whether such measures would reduce the chronic disability attached to impaired cognition in stroke in stroke patients.

First clinical researches in this field were made by Sarno and Sarno (Sarno et al., 1972a, b). Authors investigated the effect of HBOT on language skills disrupted after stroke. First study included 16 left hemisphere stroke patients, second: 32 stroke patients (16 with left sided damage). Patients underwent neuropsychological examination, with detailed assessment of various verbal processes, before chamber treatment and after every session. Each patient participated in two double blinded conditions: HBOT (performed at 2 ATA of 100% oxygen) or sham (10.5% oxygen both for 90 min) with random order. Authors found no significant changes in neuropsychological tests results after exposure to oxygen.

Boussi-Gross et al. (2015) presented a retrospective analysis of the effects of HBOT on memory impairments in poststroke patients during the late chronic, unremitting stage. The HBOT protocol consisted of 40 to 60 daily sessions, 5 days per week, 90 min each, 100% oxygen at 2 ATA. Their data showed statistically significant improvements in memory functions in the majority of patients. These neuropsychological findings were in good agreement with metabolic brain changes assessed by single photon emission computed tomography brain imaging. Imaging analysis identified the brain regions associated with the memory impairments and improvements (perirhinal cortex and its activation correlated with clinical improvement in the delayed memory measures; improvement in verbal and nonverbal delayed memory abilities with the change in activation in the left and right perirhinal cortex, respectively).

These results are consistent with previous reports that HBOT induces neuroplasticity effects at late chronic stage, although these studies lacked a control group. Nevertheless, these and previous studies have provided convincing evidence that HBOT can induce neuroplasticity at chronic poststroke stages in areas with metabolic dysfunction, which if relevant to memory function in the brain can improve after HBOT (Boussi-Gross et al., 2013; Efrati et al., 2013).

Hadanny et al. (2015) firstly examined effect of HBOT on patients suffering from anoxic brain damage caused by cardiac arrest. Patients received 60 daily HBOT sessions of 100% oxygen at 1.5 ATA for 60 min. Neuropsychological measures were compared with single photon emission computed tomography results. Authors found significant improvement in memory, attention and executive functions. Those changes correlated with increased brain activity in relevant brain areas assessed by single photon emission computed tomography imaging. Their further retrospective research focus was put on 162 stroke patients (87 in left hemisphere, 121 ischemic). They found that HBOT (40–60 sessions, 90 min of 100% oxygen at 2 ATA) had significant effect on all cognitive domains. Authors concluded that hyperbaric oxygen therapy can be successful treatment even in late chronic stage of post-stroke patients (Hadanny et al., 2020).

In one of the most recent studies, Rosario et al. (2018) measured the impact of HBOT across a number of cognitive domains, including speech, language skills, general cognition, memory and emotional/behavioural impairments. The authors assessed functional abilities over a 3-month period for 6 subjects who underwent two 4-week periods of HBOT (commenced over 6 months after a stroke)*.* HBOT comprised 20 total treatments of 100% at 2.0 ATA for 60 min for 4 weeks (on weekdays). There were significant improvements in memory and executive function after oxygen therapy. Despite a small sample size, the authors concluded that their findings support the idea of HBOT as a potential intervention following stroke. Researches on effect of HBOT on cognition after ischemia/stroke are presented in Table 5.

**Table 5:** Effect of HBOT on cognition after stroke

Authors	Studied group	HBOT protocol	Cognitive measures	Results
(Sarno et al., 1972b)	16 left hemisphere stroke patients	1 session of HBO for 90 min at 2 ATA	Token Test, Functional Communication Profile	No improvement
(Sarno et al., 1972a)	32 stroke patients (16 left sided damage)	1 session of HBO for 90 min at 2 ATA	Visual Cancellation Letters, Auditory Digit Span, Block Design, Purdue Pegboard, Two-Point Tactile Thresholds, Motor Impersistence, Token Test, Functional Communication Profile	No improvement
(Boussi-Gross et al., 2015)	91 stroke patients	40–60 daily HBOT sessions; 5/week; for 90 min at 2 ATA	NeuroTrax: immediate verbal memory, Delayed verbal memory, Immediate nonverbal memory, Delayed nonverbal memory, Total memory index	Improvement in all memory measures
(Hadanny et al., 2015)	11 patients with anoxic brain dame after cardiac arrest	60 HBOT sessions, 5 per week, for 60 min at 1.5 ATA	NeuroTrax: Verbal Memory Non-Verbal Memory, Go-No-Go task, Stroop test, Finger Tapping, Catch Game, Staged Information Processing Speed, Verbal Function and Visual Spatial Processing	Improvement in memory, attention and executive functions
(Rosario et al., 2018)	7 stroke patients	20 HBOT sessions for 60 min at 2 ATA	Boston Naming Test, Reading Comprehension Battery for Aphasia, Porch Index of Communication Ability, MMSE, California Verbal Learning Test, Grooved Pegboard Test, Trial Making Test A & B, Controlled Oral Word Association Test, Wechsler Abbreviated Scale of Intelligence- block design, Weschler Memory Scale, Delis Kaplan Executive Function System	Improvement in speech, language, memory, processing speed and executive functions
(Hadanny et al., 2020)	162 stroke patients (87 in left hemisphere, 121 ischemic)	40–60 HBOT sessions for 90 min of 100% oxygen at 2 ATA	NeuroTrax: memory, executive function, visuospatial skills, verbal function, attention, information processing speed and motor skills	Improvement in all cognitive domains

*ATA* absolute atmosphere, *HBOT* hyperbaric oxygen therapy, *MMSE* Mini-Mental State Examination

**Table 6:** Effect of HBOT on cognition in animal models of neurodegenerative disease

Authors	Studied group	HBOT protocol	Cognitive measures	Results
(Shapira et al., 2018)	14 old triple-transgenic mice and 14 non transgenic	HBOT 100% oxygen at 2 ATA for 60 min daily for 14 consecutive days	Y-maze, Open field test, Novel object recognition test	Improvement in cognition and behaviour measures
(Zhao et al., 2017)	8 rats with AD	5 HBO sessions at 2 ATA for 60 min	Morris water maze task	Improvement in learning and memory
(Zhang et al., 2010)	10 VaD rats	10 HBOT sessions for 90 min at 2 ATA	The one-way avoidance test	Improvement of learning and memory

*AD* Alzheimer Disease, *VaD* Vascular Dementia, *ATA* absolute atmosphere, *HBOT* hyperbaric oxygen therapy

Clearly, additional larger prospective, randomised trials on the effect of HBOT on cognitive impairment during acute and delayed poststroke periods should be conducted. Moreover, future studies should widen the assessment of the HBOT effects on different cognitive functions because most of the existing studies focus on memory abilities. Additional study limitations relate to the HBOT protocol. Even though they have shown similar, significant beneficial effect, studies that have evaluated HBOT in stroke management have used different treatment protocols. The exact HBOT protocol that will induce maximal neuroplasticity with minimal side effects must be determined. Bennet et al. (2014) reported that when taken together, the existing literature does not indicate that HBOT is an effective intervention in the acute phase following an ischaemic stroke. Nevertheless, the failure of some clinical stroke trials that have utilised HBOT is probably linked to factors such as delayed time to therapy, inadequate sample size and the use of excessive chamber pressures (Singhal, 2007). Further research could focus on investigating HBOT effects on cognitive functions on stroke patients receiving thrombolysis or thrombectomy treatment before oxygen therapy.

### HBOT Usage for Cognitive Ageing and Neurodegenerative Disorders

Dementia is a condition characterised by increasing several cognitive deficits such as loss of memory, problems with speech and understanding and visuo-spatial disruption. According to the 2003 World Health Organization (WHO) World Health Report, dementia causes 11.2% more years lived with disability than cardio- and cerebrovascular diseases and all forms of cancer in people aged 60 years and older. There are currently around 36 million dementia patients worldwide. It is anticipated that the number of dementia cases will increase in the subsequent years and will reach 81.1 million by the year 2040 (Ferri et al., 2005). Research on HBOT for dementia has mainly focussed on animal experiments (Table 6).

The first investigation of HBOT in the elderly population was performed by Jacobs et al. (1969). They reported improved cognitive functioning in 13 elderly patients with chronic organic brain syndrome after exposure to hyperbaric oxygen (30 sessions, twice a day with 100% oxygen at 2.5 ATA). Five control subjects exposed to a neutral air mixture failed to show improvement. Over the next six years, five other research reports were published. Three confirmed Jacobs original observation (Edwards & Hart, 1974; Jacobs et al., 1972; Raskin et al., 1978), while two did not (Goldfarb et al., 1972; Thompson et al., 1976). The study by Goldfarb et al. (1972) was performed on 10 patients (mean age 74 years) with cognitive decline. All subjects underwent 40 to 58 h of exposure to 100% oxygen at 2.5 ATA in two sessions per day (90 min each). Compression and decompression time of each individual chamber session was about 110 min. Neuropsychological examination was performed before and after 15 days of treatment. There were no significant improvements in cognitive outcome of subjects. Moreover Thompson et al. (1976) included 21 subjects with dementia (50–80 years old, 13 with diagnosis of cortical atrophy, 8 cerebrovascular disease) and 4 control subjects. HBOT consisted of the same procedure as Jacobs et al. (1969). There were no significant differences between the experimental and control subjects (Jacobs et al., 1972), although the severity of dementia in subjects in both studies was different. The patients in the Jacobs study had less cognitive deterioration. The interest in treating dementia with HBOT has grown in recent years.

Alzheimer disease is the most common form of dementia. It is characterised by progressive cognitive impairment and psychobehavioural disturbances. In a rodent model of Alzheimer disease, Shapira et al. (2018) found that HBOT can improve cognitive function by reducing neuroinflammation. The authors conducted their study with 6 mice (3 HBO treated, 3 nontreated). For animals in the treated group, HBOT was administered as 100% oxygen at a pressure of 2 ATA for 60 min daily for 14 consecutive days. The effects of HBOT on cognitive functions (memory and behaviour) in mice were evaluated using a series of behavioural tests. Cognitive tests were performed during the 7 days preceding sacrifice with a 24-h delay after the last HBOT or control treatment and a 48-h delay after the last task to reduce stress. The authors concluded that HBOT can ameliorate Alzheimer disease pathology and behavioural deficits in a transgenic mouse model of Alzheimer disease. Furthermore, in another rodent model of Alzheimer disease, Zhao et al. (2017) proved that HBOT can reduce hippocampal neuronal apoptosis and thus improve cognitive function. The authors examined 24 rats (3 groups: 8 normal, 8 Alzheimer disease, 8 Alzheimer disease HBO) that underwent the experimental procedure. The Alzheimer disease HBOT group received six HBOT sessions (2 ATA) for 60 min, while the normal and Alzheimer disease groups were placed in hyperbaric chambers without compression or decompression treatment. The authors concluded that hyperbaric treatment improves learning and memory skills by inhibiting dendritic spine loss and reducing neuronal apoptosis, astrocyte activation and tumour necrosis factor α (TNF-α) production in the hippocampus of rats with Alzheimer disease.

The second most common form of dementia is vascular dementia, accounting for approximately 30% of all cases of dementia (Kalaria et al., 2008). Vascular dementia is group of syndromes based on varying vascular mechanisms, such as multiple infarcts, small vessel ischaemic disease, strategically placed infarcts, hypoperfusion and haemorrhage, Alzheimer disease with cerebrovascular disease, hereditary vascular dementia or cerebral autosomal dominant arteriopathy with subcortical infarcts and leukoencephalopathy (Brien et al., 2003). To date, no effective treatments have been approved for established vascular dementia. Current treatment methods focus mainly on the reduction of risk factors and slowing the progression of the clinical outcome (Erkinjuntti et al., 2004; Moorhouse & Rockwood, 2008; Sorrentino et al., 2008). A study on animal models of vascular dementia demonstrated that HBOT (2 ATA for 120 min [pressure boost for 15 min, steady pressure for 90 min, decompression for 15 min] once a day for 10 continuous days) improves the blood supply and promotes neurogenesis in the piriform cortex (Zhang et al., 2010). These observations suggest possible benefits of HBOT for vascular dementia in humans.

Cerebrovascular disease includes a variety of conditions that affect the brain and the cerebral circulation which can lead to vascular dementia (Kuźma et al., 2018). Vila et al. (2005) examined possible reversible effect of HBOT on cognitive decline in cerebrovascular disease. Their study included 26 subjects with mild to moderate leukoaraiosis (18 received 10 sessions of HBOT for 45 min at 2.5 ATA, 8 air sessions at 1.1 ATA). After treatment subjects receiving hyperbaric oxygen showed significant improvement in motor and cognitive scales compared to control group.

Wang and colleagues (Wang et al., 2009) examined 64 vascular dementia patients (32 HBOT, 32 control subjects) who underwent 12 weeks of HBOT sessions (97% oxygen administered at 2.0 ATA for 60 min a day for 24 consecutive days a session; six days of rest in between sessions) as an adjuvant treatment to donepezil. Patients who received oxygen treatment showed better cognitive function compared to the control group treated with donepezil alone, measured either by the MMSE or by Hasegawa’s Dementia Rating Scale.

Similar methodology was used by Xu et al. (2019). Authors prospectively analysed 158 vascular dementia patients who were randomized in two groups: HBO treatment (12 weeks of HBOT sessions administered at 2 ATA for 60 min) and control group, both treated with donepezil hydrochloride. Authors concluded that HBO therapy can improve cognitive functions in patients with vascular dementia.

In one of the most recent reviews in this field, You et al. (2019) described HBOT as effective and safe complementary therapy for the treatment of vascular dementia. However, more research is needed because the exact mechanism of this treatment modality remains unclear. Given that HBOT has shown usefulness in treating a variety of conditions, the possible efficacy for treating Alzheimer disease and vascular dementia should be considered in experimental and preliminary clinical studies. Effect of hyperbaric oxygen therapy on dementia is presented in Table 7.

**Table 7:** Effect of HBOT on dementia

Authors	Studied group	HBOT protocol	Cognitive measures	Results
(Jacobs et al., 1969)	13 elderly patients with chronic organic brain syndrome	30 HBOT sessions, twice a day with 100% oxygen at 2.5 ATA	Wechsler Memory Scale; Bender-Gestalt Memory Ohase; Tien’s Organic Integrity Test	Improvement in cognition
(Goldfarb et al., 1972)	10 patients with cognitive decline	40 to 58 h HBOT with 100% oxygen at 2.5 ATA in two sessions per day (90 min each)	Wechsler Memory Scale; Bender-Gestalt Memory Phase; Tien’s Organic Integrity Test	No significant improvement
(Thompson et al., 1976)	21 subjects with dementia (13 with cortical atrophy, 8 cerebrovascular disease	30 HBOT sessions, twice a day with 100% oxygen at 2.5 ATA	Wechsler Memory Scale; Benton Visual Retention Test; Ravesn’s Progressive Matrices; Hooper Visual Organization Test; Tien’s Organic Tntegrity Test; Word Naming Test; Finger Tapping	No significant improvement
(Vila et al., 2005)	18 CVD patients	10 HBOT sessions for 45 min at 2.5 ATA	MMSE, Gait and Equilibrium Scale, Unified Parkinson;s Disease Rating Scale, Barthel Scale	Improvement in all scales (motor and cognition)
(Wang et al., 2009)	32 VaD patients	12 weeks of HBOT sessions 97% of oxygen at 2 ATA for 60 min	MMSE or Hasegawa’s Dementia Rating Score	Improvement in cognition
(Xu et al., 2019)	79 VaD patients	12 weeks of HBOT sessions, 5/week, 100% of oxygen at 2 ATA for 60 min	MMSE	Improvement in cognition (MMSE score)

*ATA* absolute atmosphere, *HBOT* hyperbaric oxygen therapy, *VaD* Vascular Dementia, *CVD* Cerebrovascular Disease, *MMSE* MiniMental State Examination

Future research should consider more detailed cognitive assessment. The current methodology is based mostly on brief neuropsychological batteries and tests, for example, the MMSE and MoCA. Furthermore, research must find the most effective HBOT protocol and include more subject and control groups. Moreover, multimodal assessment of positron emission tomography and magnetic resonance imaging networks could provide additional information on the impact of HBOT on neurodegenerative diseases.

## Conclusions

To the best of our knowledge this review provides the first state-of-the art, systematic summary of research focused on the use of HBOT in various neurological conditions.

Existing clinical data can be conflicting due to several inherent procedural issues, such as the use of non-objective endpoints, the lack of appropriate brain imaging as part of the inclusion criteria, inappropriate placebo of a hyperbaric environment as well as the inclusion of patients and lack of control groups. Clearly, larger randomised trials that evaluate the effect of HBOT on cognitive impairment should be conducted. Current HBOT studies have reported controversial results with regard to the efficiency of HBOT in various neurological conditions with cognitive disturbance outcome.

Some study limitations relate to HBOT itself. There is still no agreement about an HBOT protocol, specifically the appropriate air pressure and the time and repetition of treatment sessions. The exact HBOT protocol for each neurological state – which will induce maximal neuroplasticity and functional improvement with minimal side effects – must be determined.

Future studies should widen the assessment of HBOT effects on different cognitive functions because most of the existing investigations have focussed on single processes. Furthermore, more objective and precise neuropsychological assessment methods are needed to evaluate the efficacy of HBOT for neuropsychological deficits. To reach an agreement about the effectiveness of HBOT for neuropsychological disorders, research needs to focus more on homogeneity of the included subjects. The current studies were conducted using heterogeneous groups of patients with wide variations in age, severity and nature of the brain damage. Thus, there is a need for longitudinal studies to verify whether the administration of a more extensive series of HBOT sessions will lead to longer-lasting improvements in cognitive functioning.

Assuming that the methodological issues described in this review can be properly addressed and evaluated HBOT may have potential for the treatment of neuropsychological deficits in a wide range of neurological states.
